# Printable Alginate Hydrogels with Embedded Network of Halloysite Nanotubes: Effect of Polymer Cross-Linking on Rheological Properties and Microstructure

**DOI:** 10.3390/polym13234130

**Published:** 2021-11-26

**Authors:** Svetlana A. Glukhova, Vyacheslav S. Molchanov, Boris V. Lokshin, Andrei V. Rogachev, Alexey A. Tsarenko, Timofey D. Patsaev, Roman A. Kamyshinsky, Olga E. Philippova

**Affiliations:** 1 Physics Department, Moscow State University, Moscow 119991, Russia; glukhova@polly.phys.msu.ru; 2 A.N. Nesmeyanov Institute of Organoelement Compounds, Russian Academy of Sciences, Moscow 119991, Russia; bloksh@ineos.ac.ru; 3 Moscow Institute of Physics and Technology, Dolgoprudny 141701, Russia; rogachev.av@phystech.edu (A.V.R.); aleksey.spitsin@phystech.edu (A.A.T.); kamyshinsky.roman@gmail.com (R.A.K.); 4 Kurchatov Complex of NBICS-Technologies, National Research Center Kurchatov Institute, Moscow 123182, Russia; timpatsaev@mail.ru

**Keywords:** rheology, viscoelasticity, hydrogel, halloysite, alginate, network

## Abstract

Rapidly growing 3D printing of hydrogels requires network materials which combine enhanced mechanical properties and printability. One of the most promising approaches to strengthen the hydrogels consists of the incorporation of inorganic fillers. In this paper, the rheological properties important for 3D printability were studied for nanocomposite hydrogels based on a rigid network of percolating halloysite nanotubes embedded in a soft alginate network cross-linked by calcium ions. Particular attention was paid to the effect of polymer cross-linking on these properties. It was revealed that the system possessed a pronounced shear-thinning behavior accompanied by a viscosity drop of 4–5 orders of magnitude. The polymer cross-links enhanced the shear-thinning properties and accelerated the viscosity recovery at rest so that the system could regain 96% of viscosity in only 18 s. Increasing the cross-linking of the soft network also enhanced the storage modulus of the nanocomposite system by up to 2 kPa. Through SAXS data, it was shown that at cross-linking, the junction zones consisting of fragments of two laterally aligned polymer chains were formed, which should have provided additional strength to the hydrogel. At the same time, the cross-linking of the soft network only slightly affected the yield stress, which seemed to be mainly determined by the rigid percolation network of nanotubes and reached 327 Pa. These properties make the alginate/halloysite hydrogels very promising for 3D printing, in particular, for biomedical purposes taking into account the natural origin, low toxicity, and good biocompatibility of both components.

## 1. Introduction

Three-dimensional (3D) printing represents an additive technology of fabricating 3D objects by successive layer-by-layer addition of material following the digital design [1]. Of particular interest is the 3D printing of biodegradable polymers like polysaccharides, since they do not pollute the environment and can be used for biomedical purposes. Among different polysaccharides, sodium alginate (SA) is one of the most widely applied in 3D printing [2,3]. SA is a linear multiblock copolymer of 1,4-linked β-D-mannuronic (M) and α-L-guluronic (G) acids [4]. Having been extracted from the cell walls of brown algae, it is non-toxic, biocompatible, and biodegradable. Moreover, it can easily form hydrogels employed for 3D printing [2]. Most often, the alginate hydrogels are produced using multivalent cations (e.g., Ca^2+^) as cross-linkers [5]. In this case, the cross-linking was instantaneous and proceeded at very mild conditions, permitting biological objects such as living cells, enzymes, or DNA to be incorporated into the gels without damage [6]. In the cross-linking by Ca^2+^, mainly polyguluronate GG blocks of the polymer chains are involved yielding junction zones with an “egg-box” structure [7] and leaving alginate residues in other blocks without cross-linking. Such large junction zones provide rather strong cross-links while still keeping reversible character due to their non-covalent nature.

3D printing with polymer gels is one of the rapidly growing latest technologies that can be used for multiple applications, such as for fabricating complex structures to mimic native organs and functional tissues [8,9,10]. Unfortunately, 3D printed objects produced from ordinary hydrogels suffer from rather low mechanical strength. Also, insufficient viscoelasticity can deteriorate the resolution of the 3D printing as a result of the spreading of the material [11].

Several approaches were proposed to overcome the problem of the mechanical weakness of hydrogels. One of them consists of the combination of two polymers forming either double network structures [12] or interpolyelectrolyte complexes [13]. Another approach is based on the incorporation of different reinforcing fillers in the hydrogels [14,15]. In particular, alginate hydrogels were strengthened by such fillers as clay platelets [16], carbon nanotubes [17], nanofibers of polylactic acid [18], alginate nanofibrillar network [19], and some others. Nanofillers with a high aspect ratio were shown to be particularly effective for the reinforcement of the hydrogels [19] since they can enhance mechanical properties at extremely low filler loadings.

Among such fillers, halloysite nanotubes (HNTs) are of particular interest. They represent a naturally occurring clay mineral composed of aluminosilicate sheets rolled several times [20]. The inner surface (inside the lumen) is composed of aluminol groups and carries a positive charge, whereas the outer surface contains silanol groups that are negatively charged [21]. Different chemical compositions of inner and outer surfaces, as well as a large total surface area of the nanotubes, facilitate their interaction with polymer chains and, therefore, their impact on the mechanical properties of hydrogels. Halloysite was shown to be an effective reinforcing agent for various polymer hydrogels, including alginate hydrogels [22,23,24,25,26]. Moreover, due to their empty lumen, the HNTs can serve as viable nanocages for active molecules (e.g., dyes, antiseptics, etc.) [22,27] inside the hydrogel.

Although alginate is one of the most widely applied polysaccharides in 3D printing [2,3], its combination with halloysite has not yet been used for this purpose, as far as we know. We believe that the strongest enhancement of mechanical properties, upon the addition of halloysite, will occur at HNTs concentrations beyond the percolation threshold [28], when the nanotubes form their own network structure in addition to the polymeric one. Such networks of HNTs should withstand more stress at loading compared to the dispersed nanotubes and could provide the energy dissipating necessary for the hydrogel toughening [29]. The percolated structures formed by the filler are expected not only to strengthen the final 3D printed object but also to improve the printing process [3]. Therefore, in the present research, we will incorporate percolated HNTs within the alginate hydrogels.

Another factor that should affect the performance of nanocomposite hydrogels for 3D printing is the degree of the cross-linking of polymer chains. It was shown that cross-linking strengthens the system as expected [30]. At the same time, in some cases, the presence of a filler may eliminate any improvement of printability produced by cross-linking, as was shown recently in an example of the alginate/gelatin/cellulose nanocrystals system [30]. Therefore, it is important to estimate the effect of polymer cross-linking on the printability of alginate/halloysite hydrogels.

The present paper is aimed at investigating the effects of polymer cross-linking on the rheological properties and microstructure of alginate/halloysite hydrogels containing percolating halloysite nanotubes embedded in the alginate network cross-linked by calcium ions. The SAXS experiments revealed the change of the hydrogel microstructure upon cross-linking. The rheological studies demonstrated that cross-linking of polymer chains enhances the shear-thinning properties, accelerates the viscosity recovery at rest, and increases the storage modulus, but only slightly affects the yield stress, which seems to mainly be determined by the percolation network of nanotubes. Thus, for the first time, the 3D printability of the alginate/halloysite hydrogels is evaluated and they were shown to be quite a promising material.

## 2. Materials and Methods

### 2.1. Materials

HNTs from Sigma–Aldrich (Taufkirchen, Germany) were used as received. Their density was 2.53 g/cm^3^ [31] and the average specific area was 58.1 m^2^/g [32]. Sodium alginate from Sigma–Aldrich (Darmstadt, Germany), calcium chloride CaCl_2_ (>97%) from Fluka (Buchs, Switzerland), and sodium hydroxide NaOH from Acros Organics (Geel, Belgium) were used without further purification. Double distilled water purified on Milli-Q Millipore Waters (Millipore, Burlington, MA, USA) set-up was used as a solvent.

### 2.2. Preparation of Samples

A series of SA hydrogels loaded with HNTs in the presence of different amounts of cross-linker CaCl_2_ was prepared as follows. The halloysite powder was dispersed in distilled water using ultrasonic treatment for 60 min at a power of 100 W. Then SA stock solution was added, and the dispersion was stirred at 400 min^−1^ over 24 h. After that, the solution of cross-linker CaCl_2_ was poured into the dispersion and stirred at a high rotational speed of 1000 min^−1^ for another 24 h. The pH value was adjusted to 7.0–7.2 with the appropriate amount of NaOH solution. The prepared samples were left for 1 day preceding measurements for relaxation and homogenization. Alginate hydrogels (without HNTs) were prepared by mixing stock solutions of SA and CaCl_2_ at 1000 min^−1^ for 24 h. The samples retained the rheological properties for at least several months.

### 2.3. Transmission Electron Microscopy (TEM)

TEM experiments were performed on transmission electron microscope LEO912 AB OMEGA Zeiss (Oberkochen, Germany) at an acceleration voltage of 100 kV. HNTs were dispersed in distilled water using ultrasonic treatment for 30 min at a power of 100 W to prepare 0.01 wt% dilute aqueous suspension, allowing for the observation of single objects. A small droplet of the sample was put on the grid and dried at room temperature before the measurement.

### 2.4. Rheology

Rheological measurements were carried out on a stress-controlled rotational rheometer, the Physica MCR 301 (Anton Paar, Graz, Austria), using plate-plate geometry (diameter 25 mm, gap 500 μm). Temperature was maintained at 25.00 + 0.05 °C by Peltier elements. Prior to each measurement, the samples were equilibrated for 10 min after loading in the cell. The details of the measurements are described elsewhere [33,34,35].

In steady shear experiments, the dependences of viscosity on shear rate (flow curves) were measured in the range of shear rates from 0.001 to 500 s^−1^. In oscillatory shear experiments, the angular frequency dependences of the storage G′ (ω) and loss G″ (ω) moduli were measured in the linear viscoelastic regime, which was determined preliminarily by amplitude sweep tests. The variation of G′ and G″ as a function of stress amplitude was studied at a frequency of 10 rad/s. To determine the yield stress σ_y_, the shear stress sweep was applied, and shear rates were measured. To study the viscosity recovery, the rotational time sweeps were made sequentially at two different shear rates: 50 s^−1^ (during 60 s) and 0.1 s^−1^ (during 60 s).

### 2.5. IR-Spectroscopy

IR spectra were recorded on Bruker Vertex 70v FTIR spectrometer (Bruker Optic GmbH, Ettlingen, Germany) in attenuated total reflectance (ATR) mode utilizing PIKE GladyATR device with diamond ATR unit. A special clamp was used to firmly press the sample to the surface of the ATR unit. The spectra were recorded in 4000–400 cm^−1^ range with a resolution of 1 cm^−1^ for powder HNT sample and 2 cm^−1^ for gels. The spectra of hydrogels were background corrected using a spectrum of H_2_O.

### 2.6. Scanning Electron Microscopy

The morphology of the samples was examined with Versa 3D DualBeam scanning electron microscope (Thermo Fisher Scientific, Waltham, MA, USA) equipped with a Gaseous Secondary Electron Detector (GSED). For Environmental SEM (ESEM) study, the specimen was placed on a Peltier-cooled stage at 10 °C in the microscope chamber and then evacuated to a pressure of 1224 Pa. Images in ESEM mode were obtained with GSED at an accelerating voltage of 10 kV and a current of 83 pA, with the humidity decreasing from 100% to 70% to reveal morphological features of the sample. The ESEM analysis was carried out on scaffolds in a hydrated state.

### 2.7. Small-Angle X-ray Scattering (SAXS)

SAXS experiments were performed at Rigaku HighFlux HomeLab instrument (Tokyo, Japan) at the Moscow Institute of Physics and Technology (Dolgoprudny, Russia) [36]. The instrument was equipped with a pinhole camera attached to a rotating anode X-ray high-flux beam generator (MicroMax 007-HF, Tokyo, Japan) operating at 40 kV and 30 mA (1200 W). The X-ray wavelength λ was 1.54059 Å. A multiwire gas-filled detector, Rigaku ASM DTR Triton 200 (diameter of the active area was 200 mm, pixel size was ~260 μm), was placed at 2.0 m from the samples (the covered q-range was 0.006–0.19 Å^−1^) and/or 0.5 m (0.024 Å^−1^ ≤ q ≤ 0.8 Å^−1^). The azimuthal integration of the obtained 2D images was performed using the Saxsgui software (Rigaku Innovative Technologies, Inc., Tokyo, Japan) and JJ X-ray System Aps, Horsholm, Denmark). The temperature was fixed at 25 ± 0.1 °C.

## 3. Results and Discussion

### 3.1. General Consideration

For this study, we used sodium alginate with a molar mass of 220,000 g/mol (the degree of polymerization was 1250), as was determined from the intrinsic viscosity value of an aqueous solution using the Mark-Houwink equation [η] = K′ × M^a^ with the parameters K′ = 2 × 10^−3^ and a = 0.97 [2,37]. For this polymer, the entanglement concentration characterizing the transition from unentangled to entangled semidilute regime in water was equal to 0.3 wt%. As was shown by ^1^H NMR spectroscopy, the polymer contained 65 mol% G units and 35 mol% M units with the following content of sequences: 45 mol% GG, 15 mol% MM, and 40 mol% GM/MG.

**Figure 1:**
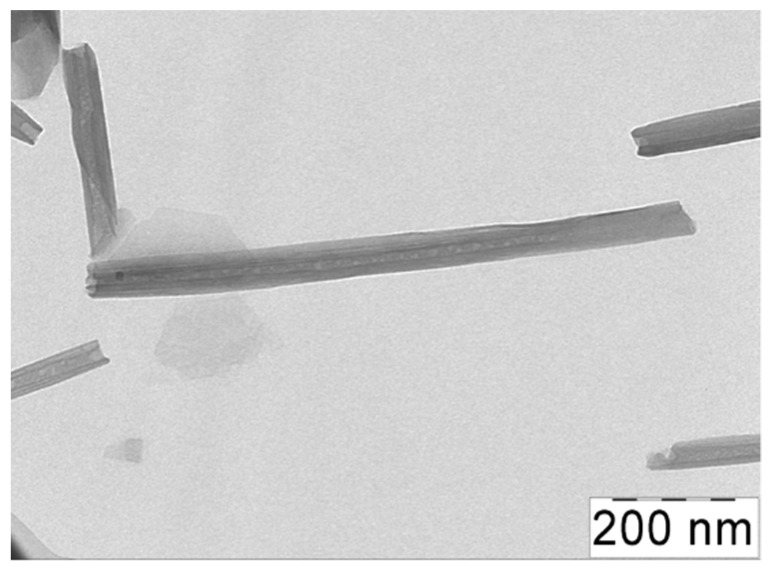
TEM micrographs of halloysite nanotubes.

The natural clay halloysite used as a filler has a chemical composition similar to kaolinite: Al_2_Si_2_O_5_(OH)·mH_2_O, where m = 2 for hydrated HNTs [38]. By TEM, it was shown that the HNTs under study had an average length L of 1000 nm and an average outer diameter D of ca. 50 nm (Figure 1), which resulted in a high aspect ratio L/D of 20. For the dispersions of the HNTs in water, the percolation concentration of the nanotubes C_HNT_* was equal to 2 vol% [39].

To study the 3D printability, a series of alginate/halloysite hydrogels differing in the concentration of calcium chloride that cross-links the polymer chains was prepared. In these samples, the concentrations of both SA and halloysite were fixed. The concentration of SA was equal to 2.7 wt% (0.15 monomol/L), which corresponded to the semi-dilute entangled regime, being almost one order of magnitude higher than the entanglement concentration (C_e_ = 0.3 wt%). The concentration of HNTs was equal to 5.4 vol%, which was 2.7-fold larger than the percolation concentration (C*_HNT_ = 2 vol% [39]). Therefore, in the resulting system, both components formed the networks.

The most promising method for the 3D printing of gels is a layer-by-layer extrusion due to its simplicity, low cost, and high printing quality [40]. In this method, certain rheological properties of the inks are required at each stage of the process [41]. The ink should shear thin to flow freely through the nozzle of the printhead and quickly recover its strong rheological properties just after deposition. The ink should possess a sufficiently high elastic modulus to keep its shape without spreading and hold the defined 3D structure. Finally, it should have a yield stress to be trapped in situ as deposited [16]. In the present study, these properties were examined to find out whether the new nanocomposite system fitted these requirements.

### 3.2. Shear-Thinning

**Figure 2:**
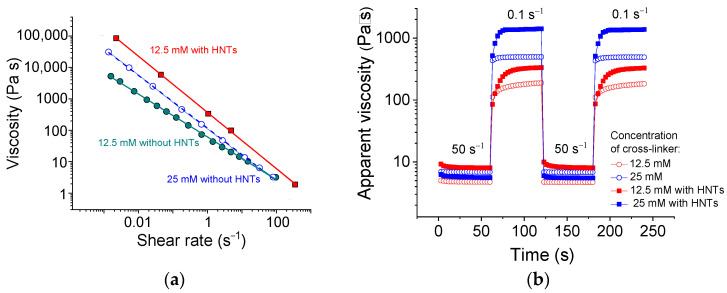
(**a**) Flow curves of 2.7 wt% sodium alginate cross-linked with 12.5 mM (cyan filled circles), or 25 mM (blue open circles) calcium chloride in water in the absence of HNTs, and with 25 mM calcium chloride in the presence of 5.4 vol% HNTs (red filled squares). (**b**) Viscosity recovery after periodic variation of shear rate (50 s^−1^ for 60 s, 0.1 s^−1^ for 60 s, etc.) for 2.7 wt% sodium alginate cross-linked with 12.5 mM (red symbols) or 25 mM (blue symbols) calcium chloride in water in the absence of HNTs (open symbols) and the presence of 5.4 vol% HNTs (filled symbols).

Figure 2a demonstrates the flow curves of SA solutions with and without HNTs. The measured viscosity values are within the applicable viscosity range for extrusion through nozzles [42]. It is seen that the system exhibits a pronounced shear-thinning behavior accompanied by the drop of viscosity η by 4–5 orders of magnitude (Figure 2a). From the slopes of these curves, the shear-thinning index n was estimated according to the formula [43]:

η = K × γ ^*n*−1^ (1)

where K is the consistency index defined as the viscosity at a shear rate of 1 s^−1^.

The values of shear-thinning index *n* thus obtained are summarized in Table 1. Note that for the uncross-linked SA solution, the shear-thinning index *n* is 0.97. Therefore, it behaves close to the Newtonian solution (*n* = 1). As seen from Table 1, for cross-linked SA, the *n* indices are much lower, indicating the shear-thinning behavior, for which *n* < 1. The *n* indices decrease from 0.3 to 0.15 with increasing concentration of cross-linker from 12.5 to 50 mM, suggesting that the shear rate destroys the alginate network. These data are consistent with the literature results showing *n* values of 0.13–0.16 [44]. Thus, the alginate cross-linked by CaCl_2_ demonstrates pronounced shear-thinning properties even in the absence of halloysite.

For alginate/halloysite hydrogels, the shear-thinning becomes more pronounced. For instance, the *n* index decreases from 0.3 to 0.07 upon the addition of 5.4 vol% HNTs to the SA cross-linked with 12.5 mM CaCl_2_. This means that the halloysite percolation network is also destroyed under shear. A similar *n* value for alginate/halloysite hydrogel (0.0754) was reported in the literature [26] as the lowest one. In the present study, the lowest shear-thinning index for alginate/halloysite is equal to 0.02. To the best of our knowledge, this is one of the smallest *n* indices observed for this system. Therefore, the present system exhibits a very pronounced shear-thinning behavior. This may be due to the breaking of the alginate/halloysite dual network as a result of the disruption of physical cross-links in the SA network and filler-filler interactions in the HNT network, as well as the alignment of polymer chains and nanotubes along the direction of flow. A similar charge of the surfaces of nanotubes [41] inducing their repulsion from each other can also facilitate the flow.

As for the consistency index, it increases significantly with increasing cross-linking of polymer chains and with increasing content of nanotubes (Table 1) since these factors enhance the viscosity at all shear rates.

The shear-thinning behavior is critically important for the systems used in injection- and extrusion-based 3D printing at the stage when the ink is driven through a nozzle. This process occurs at shear rates of 50–300 s^−1^ [42,45,46]. From Figure 2a, one can see that at 50 s^−1^, the viscosity of alginate/halloysite dual networks drops almost by four orders of magnitude compared with low shear rate data. So pronounced shear-thinning should significantly facilitate the extrusion.

### 3.3. Viscosity Recovery

Being deposited after extrusion, the ink should promptly rebuild its structure and regain its strong rheological properties. To check this behavior, the recovery of viscosity with time after a sudden change in the applied shear rate was investigated (Figure 2b). For this experiment, two shear rates (50 and 0.1 s^−1^), simulating respectively the conditions at extrusion and at rest, were used. Upon application of shear rate 50 s^−1^, the viscosity dropped by more than two orders of magnitude, whereas upon switching the shear rate to 0.1 s^−1^, the viscosity recovered. A fast decrease of viscosity upon application of the shear rate of 50 s^−1^ indicated fast destruction of both networks. Prompt recovery suggested a quick rebuilding of the dual network structure.

From Figure 2b, one can see that upon the drop of the shear rate to 0.1 s^−1^, the viscosity grew by 1.5 orders of magnitude within 3 s only and reached the required minimum viscosity at that stage of 3D printing—100 Pa·s [41]. At the same time, 96% of the viscosity was restored in 18 s (for the sample with 25 mM CaCl_2_). This was much faster than in the case of alginate/graphene oxide gels, which recovered 80% of their viscosity in 20–30 s [45].

Note that the SA networks without HNTs completely recovered their viscosity much faster (in 3–6 s) than the dual network hydrogels (18 s) (Figure 2b), but their final viscosity was much smaller. Figure 2b shows that 3 s after the switching of the shear rate to 0.1 s^−1^, the dual network had the same viscosity as the single SA network, indicating that the SA network recovered first and that the remaining 15 s were necessary to rebuild the second network. Therefore, the percolated structure of the HNTs recovered slower than the structure of the polymer network.

It is important that the hydrogels under study restore their initial viscosity completely after several cycles of shear rate switching (Figure 2b). Therefore, the nanotubes do not hinder a total (100%) rebuilding of the network structure. This is an important advantage of this system, taking into account that the filler may inhibit a complete regain of viscosity. This was, in particular, observed in alginate/graphene oxide hydrogels [45], where the viscosity was recovered only by 85.5%.

From Figure 2b, it is apparent that, with an increasing amount of cross-linker, the rate of viscosity recovery increased. For instance, 90% of viscosity was recovered in 27 and 12 s for the samples with 12.5 and 25 mM CaCl_2_, respectively. This may have been due to the fast diffusion of small cross-linker ions favoring prompt recovery. One can expect that the addition of larger amounts of cross-linker will further accelerate the recovery rate.

Thus, the alginate/halloysite hydrogels under study could regain high enough viscosity for 3D printing (100 Pa·s) in a few seconds and completely recover their viscosity of up to 1400 Pa·s in 18 s.

### 3.4. Storage Modulus

**Figure 3:**
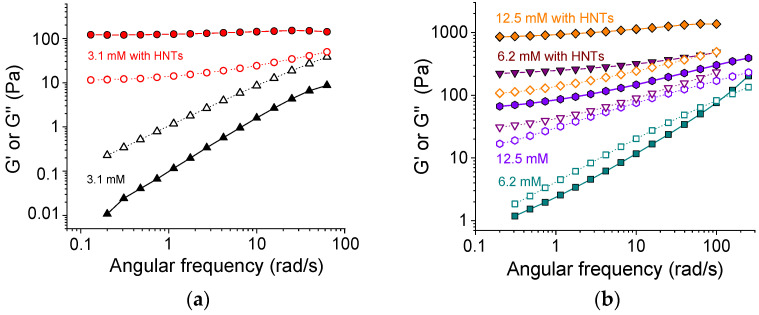
Frequency dependencies of storage G′ (filled symbols) and loss G″ (open symbols) moduli for 2.7 wt% solutions and gels of sodium alginate at different concentrations of cross-linker calcium chloride without HNTs ((**a**) 3.1 mM (black triangles), (**b**) 6.2 mM (cyan squares), and 12.5 mM (violet hexagons)), and with 5.4 vol.% HNTs ((**a**) 3.1 mM (red circles), (**b**) 6.2 mM (purple triangles), and 12.5 mM (orange diamonds)).

Figure 3 demonstrates the evolution of storage G′ and loss G″ moduli upon addition of HNTs to SA networks with different content of cross-linker. From Figure 3a, one can see that at a low amount of cross-linker (3.1 mM CaCl_2_ or 1 calcium ion per 48 alginate repeat units), the effect of added HNTs was very big. Without nanotubes, the loss modulus G″ was higher than the storage modulus G′ in all studied frequency range. Therefore, the system demonstrated a fluid-like behavior. Upon the addition of 5.4 vol.% nanotubes, the situation was reversed. The storage modulus G′ became higher than the loss modulus G″ at all studied frequencies. Moreover, G′ became independent of frequency. This indicated that the nanotubes induced the transition from a liquid-like to a gel-like system. Therewith, the value of the storage modulus increased by up to four orders of magnitude upon the addition of halloysite. These data suggest that the HNTs formed a percolation network, which is responsible for the observed gel-like behavior.

At a higher amount of cross-linker (6.2 mM CaCl_2_), the SA solution without HNTs demonstrated the rheological properties of a viscoelastic fluid: the loss modulus exceeded the storage modulus G″ > G′ at low frequencies and the storage modulus became larger than the loss modulus G′ > G″ at high frequencies (Figure 3b). Upon the addition of nanotubes, the storage modulus G′ increased by up to two orders of magnitude and became higher than the loss modulus G″ in the whole studied frequency range. Also, it became almost independent of frequency, demonstrating a large viscoelastic plateau G_0_ (Figure 3b). This indicated the formation of the network structure. The G_0_ value grew from 130 to 270 Pa at a two-fold increase of the amount of cross-linker (from 3.1 to 6.2 mM), suggesting that both components (SA and HNTs) contributed to the elasticity.

At an even higher amount of cross-linker (12.5 mM CaCl_2_) for the polymer alone (without HNTs), the elastic properties prevailed the viscous ones (G′ > G″) in the whole studied range of frequencies (Figure 3b). So, at this cross-linker concentration corresponding to 1 calcium ion per 12 polymer units, the alginate alone formed a gel. The addition of HNTs induced the increase of the moduli G′ and G″ by one order of magnitude. Thus, at 12.5 mM CaCl_2_, a dual network was formed.

At 12.5 mM CaCl_2_, the G_0_ for alginate/halloysite hydrogel was ca. 1160 Pa, whereas the G_0_ for the corresponding SA gel without nanotubes was ca. 90 Pa (at frequencies below 10 rad/s). Thus, the HNTs increased the plateau modulus by more than 1000 Pa. This input of the halloysite network was much higher than in the sample with a minimal amount of cross-linker (3.3 mM), when the polymer network was not yet formed (G_0_ = 130 Pa). This may indicate that the cross-linking of polymers may strengthen the HNTs network as well. One of the possible reasons for such behavior is as follows. HNTs are expected to interact with alginate chains since they contain surfaces (the interior of the nanotubes and the edges [47]) that are oppositely charged with respect to polymers. This interaction results in the electrostatic binding of SA to the nanotubes. H-bonds can also contribute to the binding [26]. The FTIR spectra (Figure S1) show a displacement of the peak of symmetric stretching vibrations of COO^–^ groups of SA to higher wavenumbers from 1414 to 1418 cm^−1^ in the presence of HNTs. Such behavior was previously observed by Huang et al. [26] and was interpreted as an indication of hydrogen bonding between SA and HNTs. As a result of these interactions, 0.097 g SA were adsorbed on 1 g of halloysite, as demonstrated by SANS [48]. One can expect that calcium ions are able to cross-link not only free SA chains but also SA chains bound to nanotubes. This should result in the strengthening of the network of percolating HNTs.

**Figure 4:**
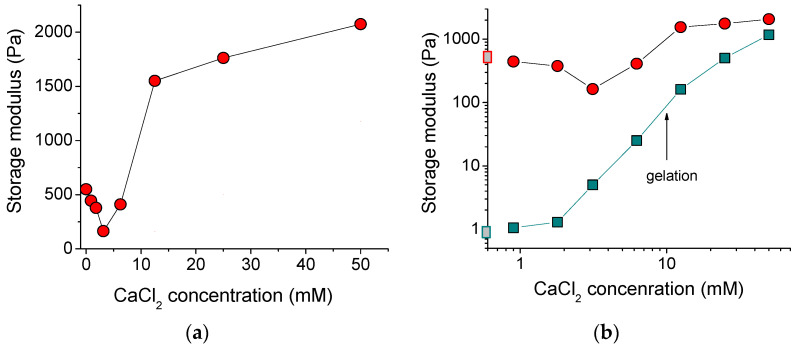
(**a**) Storage modulus as a function of concentration of cross-linker calcium chloride for 2.7 wt% solutions and gels of sodium alginate containing 5.4 vol% HNTs. (**b**) Log-log dependence of storage modulus at frequency of 50 rad/s on the concentration of cross-linker calcium chloride for 2.7 wt% solutions and gels of sodium alginate in the absence of HNTs (cyan squares) and the presence of 5.4 vol% HNTs (red circles). Gray squares on the *y*-axis denote the storage modulus in the absence of cross-linker. The arrow indicates the gelation conditions for alginate solution (without halloysite).

Figure 4a shows the dependence of the storage modulus G′ of the alginate/halloysite system on the concentration of polymer cross-linker. At low amounts of added cross-linker, the G′ dropped, but further addition of CaCl_2_ enhanced the storage modulus up to the values (2070 Pa), which were 4-fold higher than for the initial uncross-linked alginate/halloysite system (550 Pa). The initial drop of storage modulus may have been due to an extremely low amount of CaCl_2_, which was insufficient to cross-link most of the polymer chains to form a network occupying the whole volume of the system. Partial cross-linking of some SA macromolecules resulted in the formation of microgels, which may not have contributed to the elasticity of the total system. The minimum value of storage modulus was observed at the addition of 1 calcium ion per 48 repeat units of the alginate chains. A pronounced increase of the storage modulus occurred between 6.2 and 12.5 mM CaCl_2_, where the gelation in the whole system proceeded as followed by the dynamic rheology data (Figure 3b).

Figure 4b allows comparison of the storage moduli G′ for the alginate/halloysite system with alginate alone (without halloysite). The storage moduli for the alginate/halloysite system were always higher, indicating that HNTs increase the elastic properties. The most pronounced impact of nanotubes was observed for the uncross-linked alginate solution. For this system, 5.4 vol% HNTs induced the increase of the storage modulus from 1 to 550 Pa. For the cross-linked system, the effect of the nanotubes on G′ was smaller and decreased with an increasing amount of cross-linker. At the highest concentration of cross-linker used in this study (50 mM), the nanotubes increased the storage modulus from 1177 to 2074 Pa. So, at this high amount of cross-linker, the input of the polymer network in the elasticity of the whole system became as strong as that of the halloysite network.

From Figure 4b, it is evident that the increase of G′, denoting the onset of cross-linking of the pure alginate system, started at 2 mM of calcium ions. In the case of the alginate/halloysite system, the increase of G′ was observed at somewhat higher CaCl_2_ concentration (3 mM). This may indicate that the HNTs hindered the cross-linking of the SA chains. This may be related to the adsorption of some of the alginate chains on the nanotubes. Indeed, in the dispersion containing 5.4 vol% nanotubes, 1.2 wt% SA should have been adsorbed on the filler, thereby decreasing the concentration of free SA in the solution from 2.7 to 1.5 wt%. A smaller concentration of free alginate chains in the solution reduced the number of polymer-polymer contacts, thereby decreasing cross-linking.

Thus, the nanotubes reinforced the hydrogel by increasing its storage modulus up to 2 kPa, which will strengthen the 3D-printed objects.

### 3.5. Yield Stress

**Figure 5:**
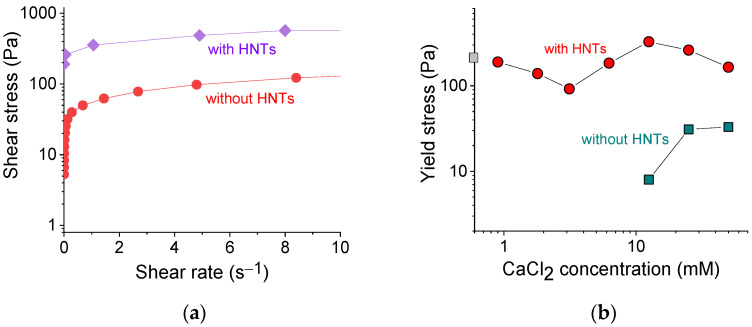
(**a**) Shear stress as a function of shear rate for 2.7 wt% sodium alginate cross-linked with 12.5 mM calcium chloride in the absence (red curve) and presence of 5.4 vol% HNTs (violet curve). (**b**) Log-log dependence of the yield stress on the concentration of cross-linker calcium chloride for 2.7 wt% sodium alginate in the absence of HNTs (cyan squares) and the presence of 5.4 vol% HNTs (red circles). Gray square on the *y*-axis denotes the yield stress without cross-linker.

Figure 5a shows the typical dependences of shear stress σ on shear rate $\dot{\gamma }$ for SA hydrogels with and without HNTs. Both gels possessed a yield stress σ_y_, indicating the limit of the elastic behavior: below it, the sample did not flow; above it (σ > σ_y_), the flow began, which is reflected in a significant increase of the shear rate as a result of the gel destruction. The yield stress values were estimated from the σ ($\dot{\gamma }$) curves by fitting them with the Herschel-Bulkley equation [49].

(2) $$\sigma =\sigma_{y}+K\cdot {\dot{\gamma }}^{n}$$

Figure 5b shows the dependence of the yield stress σ_y_ values thus obtained on the concentration of the cross-linker of polymer chains. In the pure alginate (without HNTs), the yield stress appeared only at 12.5 mM CaCl_2_, just after gelation. The σ_y_ values further increased with an increasing concentration of cross-linker from 12.5 to 25 mM, which was due to the formation of a denser network.

In the alginate/halloysite system, the polymer cross-linking led first to a lowering of σ_y_ values and then to their increase, which reflected the evolution of G′ (Figure 4b), discussed above. But in contrast to G′, one cannot observe a significant improvement of σ_y_ with increasing polymer cross-linking. Indeed, the σ_y_ values without cross-linking (175 Pa) and in the presence of 50 mM CaCl_2_ (165 Pa) did not differ significantly. Therefore, the cross-linking did not affect appreciably the yield stress values of the nanocomposite gel.

At the same time, these values were significantly enhanced upon the addition of HNTs. For instance, for 2.7 wt% SA cross-linked with 12.5 mM CaCl_2_, the yield stress increased from 8 to 327 Pa upon the addition of 5.4 vol% HNTs. A significant increase of yield stress in the presence of HNTs can be due to the formation of the second network composed of nanotubes and its strengthening by an adsorbed polymer. Thus, noncovalent filler-filler, as well as filler-polymer interactions allow the rigid skeleton of nanotubes to form a stable percolated structure that behaves akin to elastic solid below a yield stress. The yield stress value observed in the present system was rather high for hydrogel inks. For instance, it was 20-fold larger than for alginate/gelatin gel [50] and was close to the yield stress in PEG/laponite system [41].

**Figure 6:**
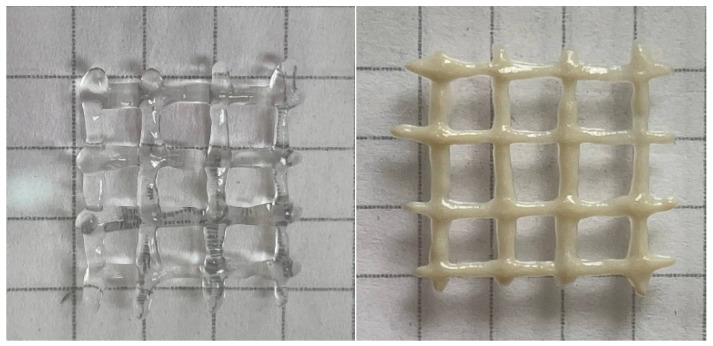
3D printed crosshatch structures made from gel containing 2.7 wt% sodium alginate and 25 mM CaCl_2_ in the absence of HNTs (**left**) and the presence of 5.4 vol% HNTs (**right**). The structures were extruded from a 0.4 mm nozzle at shear rate of 5 mm/s.

The yield stress is very important for 3D printing. It allows the ink to immediately fix its shape and then build up its strength. Otherwise, the gels remain fluid for some time after extrusion [19] and fail to fix their shape properly. This effect can be seen in Figure 6, where the hydrogels with HNTs that possessed a high yield stress demonstrated better print fidelity.

Thus, it was shown that the polymer cross-linking played an important role in the printability of alginate/halloysite hydrogels, since it enhanced the shear-thinning properties, accelerated the viscosity recovery at rest, and increased the storage modulus. At the same time, the yield stress was only slightly affected by the polymer cross-linking since it was provided mainly by the network of percolating nanotubes.

### 3.6. Microstructure

**Figure 7:**
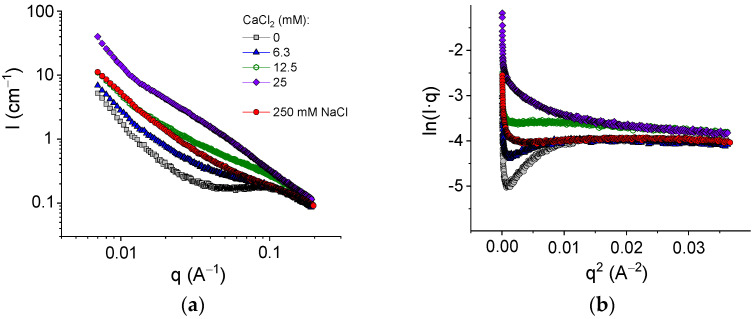
(**a**) SAXS profiles for samples containing 2.7 wt% sodium alginate and different amounts of CaCl_2_: 0 mM (gray squares); 6.3 mM (blue triangles); 12.5 mM (green open hexagons); and 25 mM (violet diamonds); and for 2.7 wt% sodium alginate in the presence of 250 mM NaCl in water. (**b**) Guinier plots ln(Iq) vs. q^2^ for the same samples.

The structure of the networks was studied by SAXS. Figure 7a shows the evolution of the scattering curves with an increasing amount of cross-linker for pure alginate in the absence of HNTs. For the initial SA solution without a cross-linker, one can see a broad peak around 0.1 Å^−1^. A similar peak was previously described for SA solutions [50] and attributed to a correlation, which arose from electrostatic repulsion between similarly charged polymer chains. In the presence of 250 mM NaCl, the peak disappeared because of the screening effect of salt (Figure 7a).

Figure 7a shows that upon cross-linking with calcium chloride, the scattering intensity drastically increased. This was due to chain associations with the formation of junction zones involving GG blocks [51]. Simultaneously, the correlation peak disappeared as a result of the screening of the electrostatic repulsion by salt (CaCl_2_).

The formation of junction zones should be reflected in the thickness of the polymer chains, which can be estimated from SAXS data. Alginate has a long persistence length of about 15 nm [52,53], and, therefore, at a small length scale, the local structure of alginate chains can be approximated as rod-like, as was demonstrated previously [51,54]. The scattering function for such a structure can be described with the Guinier approximation [54]:

$$\mathrm{qI}\left(q\right)\approx \exp\left(-R_{g,c}^{2}\;q^{2}/2\right)$$

where R_g,c_ is the cross-sectional radius of gyration. The values of R_g,c_ can be obtained from the slope of the linear fragment of the Guinier plots (Figure 7b), representing the scattering data as ln(Iq) vs q^2^. To avoid the contribution of the structure peak, the experiments with the SA solution in the absence of the cross-linker were performed in 250 mM NaCl (Figure 7b). At these conditions, the estimation gave the value of the cross-sectional radius of gyration of alginate chains of 2.8 ± 0.2 Å (Table 2), which is consistent with the data previously reported for uncross-linked alginate (2.9–3.6 Å [51,54]). Upon addition of the cross-linker, the R_g,c_ values increased, reaching 5.6 Å at 25 mM CaCl_2_ (Table 2), which was twofold higher than the radius of a single chain. According to Stokke et al. [54], thicker rod-like structures indicated lateral aggregation of GG-blocks cross-linked by calcium ions. Therefore, the guluronic sequences were paired up at gelation, which was in accordance with the “egg-box” model of the junction zones in calcium cross-linked alginate [7]. Thus, cross-linking of GG blocks of alginate chains by calcium ions was accompanied by the formation of junction zones composed of two laterally arranged chain fragments. Such junction zones made the cross-links stronger and led to pronounced enhancement of the viscoelastic properties.

**Figure 8:**
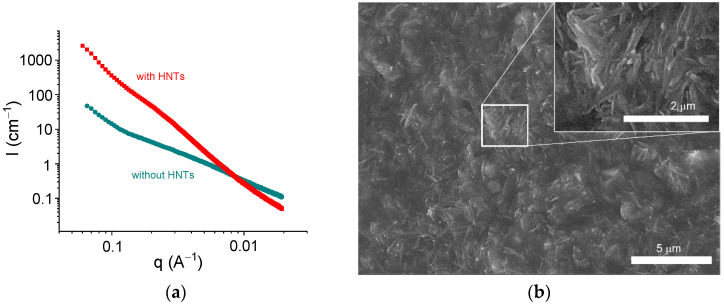
(**a**) SAXS profiles for samples containing 2.7 wt% sodium alginate and 25 mM CaCl_2_ without HNTs (circles) and with 5.4 vol% HNTs (squares) in water. (**b**) ESEM picture for sample containing 2.7 wt% sodium alginate and 25 mM CaCl_2_ with 5.4 vol% HNTs.

Figure 8 shows the scattering curves for alginate hydrogels in the presence and absence of halloysite. The addition of HNTs significantly increased the scattering intensity at q < 0.08 Å^−1^ and induced the appearance of an upturn at very low q (0.015 Å^−1^). Similar curves were previously obtained for alginate/halloysite hydrogels by Cavallaro et al. [31]. The upturn at a very low q was considered as an indication of the presence of a percolating network of HNTs [31]. Strong scattering from HNTs did not permit a revelation of the structural changes provided by the cross-linking of polymer chains, but one can expect that the cross-linking proceeded in the same way as for pure alginate hydrogels, that is, by the formation of junction zones composed of two laterally arranged chain fragments. The structure of the alginate/halloysite hydrogel was visualized by ESEM in a hydrated state (Figure 8b). The picture confirms that the nanofiller was well dispersed inside the matrix and formed a percolating structure.

Thus, the SAXS and ESEM data indicate the presence of a percolating network of HNTs in the alginate/halloysite hydrogels and reveal the formation of junction zones, including two laterally arranged chain fragments in the alginate hydrogels. Both these structures were responsible for the enhancement of the rheological properties.

## 4. Conclusions

In the present paper, rheological studies were performed in order to evaluate the printability of alginate/halloysite hydrogels with different degrees of cross-linking. The hydrogels were based on a dual network structure consisting of a rigid network of percolated clay nanotubes embedded in a soft network of alginate chains cross-linked by calcium ions. It was shown that the system possessed highly pronounced shear-thinning behavior, leading to a drop of viscosity by 4–5 orders of magnitude, which was essential for providing the flow of the system through the nozzle. At rest, the viscosity quickly recovered, reaching 100 Pa·s in 3 s and up to 1400 Pa·s in 18 s. A complete (100%) regain of the initial viscosity was observed after several cycles of shear rate switching. This indicated the ability of the hydrogel to promptly recover its strong rheological properties after extrusion and be reused several times if necessary.

The storage modulus G′ of the hydrogel was highly dependent on the concentration of the cross-linker. At low CaCl_2_ concentrations of up to 3 mM, the storage modulus decreased upon polymer cross-linking, which was ascribed to the formation of microgels that do not contribute to the elasticity of the whole system. However, further increasing of CaCl_2_ concentration resulted in a significant enhancement to the modulus that reached the value of 2074 Pa, which was 4-fold higher than for the initial uncross-linked alginate/halloysite system (550 Pa). A pronounced effect of cross-linking on the gel elasticity could be due to the formation of large junction zones involving two laterally arranged chain fragments in the alginate chains, as was demonstrated by SAXS structural studies. Enhanced elasticity was important for strengthening the 3D printed objects.

The alginate/halloysite hydrogels exhibited a yield stress of up to 327 Pa, which is necessary to fix the shape of a 3D printed construct just after deposition. Note that the cross-linking did not appreciably affect the yield stress values of the nanocomposite gel, which were determined mainly by a rigid network of percolating HNTs that behaved similarly to elastic solid below a yield stress. The formation of this network was confirmed by SAXS and ESEM.

Thus, using the network of percolating HNTs as a filler together with the proper cross-linking of polymer chains improved the rheological properties of alginate/halloysite hydrogels so that they fulfilled the requirements for the inks designed for extrusion-based 3D printing. Taking into account the natural origin and biocompatibility of both components of alginate/halloysite hydrogels, such systems are extremely promising in manufacturing ecologically friendly 3D constructs for various purposes, for instance, for soft robotics and flexible electronics.

## Figures and Tables

**Table 1:** Consistency and shear-thinning indices for 2.7 wt% sodium alginate cross-linked by calcium chloride in water at different concentrations of cross-linker in the presence and absence of halloysite nanotubes.

Concentration of HNTs, vol%	Concentration of CaCl_2_, mM	Consistency Index K, Pa·s^n^	Shear-Thinning Index *n*
0	12.5	55 ± 5	0.30 ± 0.01
0	25	76 ± 7	0.16 ± 0.01
0	50	112 ± 11	0.15 ± 0.01
5.4	3.1	100 ± 9	0.03 ± 0.01
5.4	6.2	110 ± 10	0.02 ± 0.01
5.4	12.5	335 ± 30	0.07 ± 0.01

**Table 2:** The cross-sectional radii of gyration for alginate chains cross-linked by calcium chloride in 2.7 wt% aqueous solutions and gels of sodium alginate.

Concentration of CaCl_2_, mM	Cross-Sectional Radius of Gyration R_g,c_, Å
0 *	2.8 ± 0.2
6.3	3.3 ± 0.3
12.5	4.2 ± 0.1
25	5.6 ± 0.1

* In 250 mM NaCl.

## Data Availability

The data presented in this study are openly available.

## Supplementary Materials

The following are available online at https://www.mdpi.com/article/10.3390/polym13234130/s1: Figure S1, ATR-FTIR-spectra of alginate/halloysite hydrogel (green curve) containing 2.7 wt% sodium alginate cross-linked with 25 mM calcium chloride in water and 5.4 vol% halloysite, of alginate hydrogel (black curve) containing 2.7 wt% sodium alginate cross-linked with 25 mM calcium chloride in water and of halloysite powder (blue curve).
