# Supplementary material for: Printable Alginate Hydrogels with Embedded Network of Halloysite Nanotubes: Effect of Polymer Cross-Linking on Rheological Properties and Microstructure
Source: Polymers (Basel). 2021 Nov 26;13(23):4130. doi: 10.3390/polym13234130 (PMC8659288; doi:10.3390/polym13234130)
Supplement: Supplementary file 1 [file polymers-13-04130-s001.zip › polymers-1471995-supplementary.pdf]

polymers

MDPI

Supplementary Data

Printable Alginate Hydrogels with Embedded Network of Halloysite Nanotubes: Effect of Polymer Cross-Linking on Rheological Properties and Microstructure

Svetlana A. Glukhova [1], Vyacheslav S. Molchanov [1,*], Boris V. Lokshin [2], Andrei V. Rogachev [3], Alexey A. Tsarenko [3], Timofey D. Patsaev [4], Roman A. Kamyshinsky [3,4] and Olga E. Philippova [1,*]

1 Physics Department, Moscow State University, Moscow 119991, Russia; glukhova@polly.phys.msu.ru
2 A.N. Nesmeyanov Institute of Organoelement Compounds, Russian Academy of Sciences, Moscow 119991, Russia; bloksh@ineos.ac.ru
3 Moscow Institute of Physics and Technology, Dolgoprudny 141701, Russia; rogachev.av@phystech.edu (A.V.R.); aleksey.spitsin@phystech.edu (A.A.T.); kamyshinsky.roman@gmail.com (R.A.K.)
4 Kurchatov Complex of NBICS-Technologies, National Research Center Kurchatov Institute, Moscow 123182, Russia; timpatsaev@mail.ru
* Correspondence: molchan@polly.phys.msu.ru (V.S.M.); phil@polly.phys.msu.ru (O.E.P.)

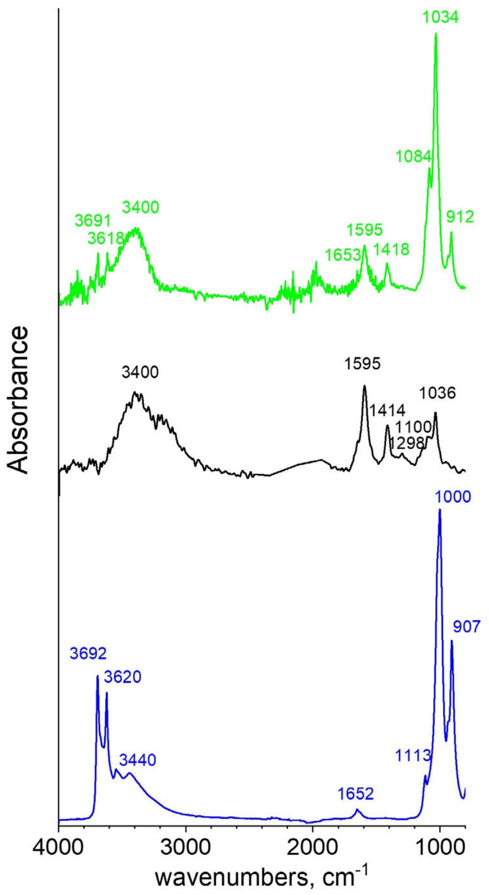

Figure S1. ATR-FTIR-spectra of alginate/halloysite hydrogel (green curve) containing 2.7 wt% sodium alginate cross-linked with 25 mM calcium chloride in water and 5.4 vol% halloysite, of alginate hydrogel (black curve) containing 2.7 wt% sodium alginate cross-linked with 25 mM calcium chloride in water and of halloysite powder (blue curve).
